# Charge Transfer Effect on Raman and Surface Enhanced Raman Spectroscopy of Furfural Molecules

**DOI:** 10.3390/nano7080210

**Published:** 2017-08-02

**Authors:** Fu Wan, Haiyang Shi, Weigen Chen, Zhaoliang Gu, Lingling Du, Pinyi Wang, Jianxin Wang, Yingzhou Huang

**Affiliations:** 1 State Key Laboratory of Power Transmission Equipment & System Security and New Technology, Chongqing University, Chongqing 400044, China; shihaiyang@cqu.edu.cn (H.S.); weigench@cqu.edu.cn (W.C.); gzl1989619@163.com (Z.G.); dulingling2014@163.com (L.D.); wangpingyi@cqu.edu.cn (P.W.); wangjianxin16@163.com (J.W.); 2 Soft Matter and Interdisciplinary Research Center, College of Physics, Chongqing University, Chongqing 400044, China

**Keywords:** surface-enhanced Raman spectroscopy, density functional theory, furfural-M*_x_* complexes, transformer aging

## Abstract

The detection of furfural in transformer oil through surface enhanced Raman spectroscopy (SERS) is one of the most promising online monitoring techniques in the process of transformer aging. In this work, the Raman of individual furfural molecules and SERS of furfural-M*_x_* (M = Ag, Au, Cu) complexes are investigated through density functional theory (DFT). In the Raman spectrum of individual furfural molecules, the vibration mode of each Raman peak is figured out, and the deviation from experimental data is analyzed by surface charge distribution. In the SERS of furfural-M*_x_* complexes, the influence of atom number and species on SERS chemical enhancement factors (EFs) are studied, and are further analyzed by charge transfer effect. Our studies strengthen the understanding of charge transfer effect in the SERS of furfural molecules, which is important in the online monitoring of the transformer aging process through SERS.

## 1. Introduction

Compared to existing detection methods, Raman spectroscopy is one of the spectral analysis techniques based on the Raman effects, which can quickly complete non-contact and non-destructive detection by Laser Scanning Confocal Microscopy (LSCM) [1,2,3,4]. The vibration of molecules in different ways (atomic swing, twisting and vibration of chemical bonds) is caused by the transfer of energy under the irradiation of a laser, and is accompanied by low frequency light generation [5,6]. The frequency changes depend only on the characteristics of the scattering material, and the vibrational spectrum is unique for different molecules. Thus, it can be used to identify the molecular species of substances, also known as fingerprint spectra. However, the disadvantages of lesser molecular scattering cross sections have led to applications being hindered [7,8,9]. Therefore, the most difficult problem at this stage remains how to effectively enhance the Raman scattering signal. The Raman intensity of pyridine adsorbed on the surface of a rough silver electrode was nearly 10^6^ times higher than the signal of the same concentration in the solution found by Albrecht et al. and Van Duyne et al. [10,11,12]. This enhancement was known as surface-enhanced Raman scattering (SERS) spectra, which result from two kinds of mechanisms. One of those is the electromagnetic (EM) enhancement mechanism, in which the laser induced the collective oscillation of electrons, resulting in the enhancement of the local electromagnetic field, leading to an increase in Raman signals [13,14,15,16]. On the other hand, Chemical Enhancement (CE) has always been present and complicated. CE results from the change of external environment; that is, interference factors lead to changes in molecular structure and redistribution of electrons, where select Raman peaks are selectively enhanced enormously. Typically, the chemical enhancement is explained via the charge-transfer mechanism [17,18,19].

Furfural is a furan derivative in which the hydrogen atom is replaced by an aldehyde group, also called furan formaldehyde [20]. In the process of transformer aging, the total source of furfural is the hydrolysis ring of cellulose molecules (the component of insulating paper) in the pyrolysis reaction [21,22]. Therefore, the content of furfural directly reflects the degree of aging of the instrument. The monitoring of furfural concentration in transformer oil can effectively reflect the operation of transformers. In addition, liquor flavor is also related to the concentration of furfural. It is very important for liquor production and scientific research to study the relationship between aroma substances and flavor types in liquor [23,24,25]. Overall, the study of furfural has great application value.

In this work, in order to study the mechanism of chemical enhancement of metal clusters, the DFT method was used to optimize molecular configurations of furfural and furfural-M_*x*_ complexes. The optimized geometries and calculated Raman spectra of the complexes and furfural were obtained. The calculated Raman spectrum of furfural was in good agreement with the experimental results, except there was a distinct difference peak at 1726 cm^−1^. The main mechanism for the difference is that there are many electron–hole pairs near the C=O bond, which makes it more easily disturbed by external factors. And the static potential distribution map also confirms the results. In addition, the Raman activities of peak 1726 cm^−1^ becomes larger with the increase of Ag atoms. The redistribution of charge leads more and more electrons to transfer to silver clusters, and the polarizability of molecules increases. It is also found that the blue shift of the characteristic peaks at 1726 cm^−1^ is related to the change of the C=O chemical bonds. Further studies have also found the influence of atomic species on the Raman activities. The simulation results show that the SERS effects of silver atoms are mostly outstanding, and consistent with the experiments. Moreover, the offset of the peak position always corresponds to the change of C=O bond length for different metal atom. These results further illustrate the effect of chemical enhancement on surface enhanced Raman scattering.

## 2. Results and Discussion

**Figure 1:**
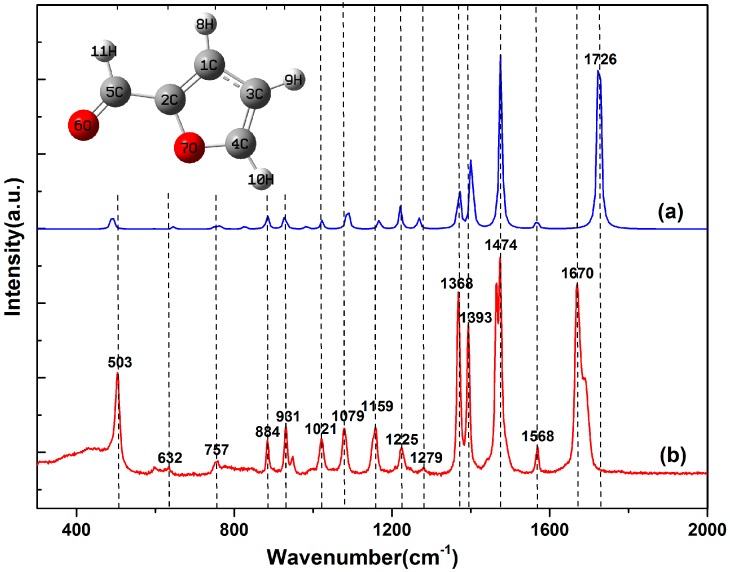
Raman spectra of pure furfural molecules: (**a**) Calculations and (**b**) Experimental measurements. The inset depicts the molecular structure of furfural molecule.

**Figure 2:**
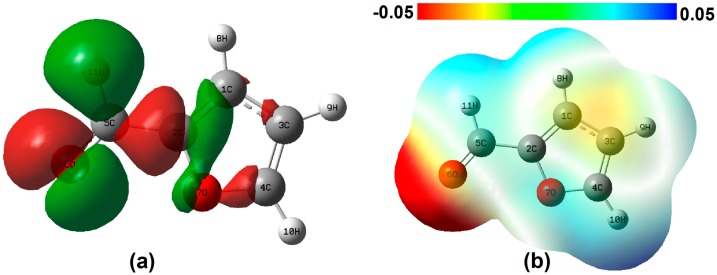
(**a**) Charge difference densities of furfural (the green and red stand for holes and electrons, respectively); (**b**) The static potential distribution of furfural (the red and blue represent low potential and high potential, respectively).

The simulated Raman spectra of single furfural molecules are shown in Figure 1 (blue line). As we can see, the virtual frequency is not found in the calculation results, which shows that the optimized molecular structure is stable. In addition, Figure 1 shows the Raman spectrum of the pure furfural solution by experimental detection (red line). In the whole Raman shift, vibration modes at 1366, 1393, 1474 and 1670 cm^−1^ predominate in the spectrum. By comparing the theoretical and experimental results, the relative intensities and Raman shift of spectra are approximately consistent when the correction factor is 0.98. However, there are still some slight differences. From the macro perspective, the main reason causing this difference is the simulation of the vibration spectra of single furfural molecule, without considering the interaction between the molecules, but the SERS experimental measurements is based on lots of molecule and in a complicated ambient environment [26], indicating the presence of intermolecular forces. In order to understand the specific micro mechanism, the Raman bands were identified for various vibrational modes based on the results of DFT, as shown in Table 1. Specifically, the difference of the characteristic Raman shift is less the 10 cm^−1^, except the peak at 1726 cm^−1^. In Table 1, the peak at 1726 cm^−1^ is mainly influenced by the asynchronous stretch of bond C=O. Hence, the reason may be attributed to the easy hydration reaction between the molecules at C=O, according to the references. However, the reasons for the molecular properties are more compelling. In Figure 2a, charge difference densities indicate that many electron-hole pairs are distributed around the C=O bonds, so the chemical bond is more susceptible to external factors. In addition, the static potential distribution (SPD) also confirms this point in Figure 2b. Red and blue areas represent the electron-rich region with electrophilic activity and electron deficient region with nucleophilic activity, respectively. For the red, the electrostatic potential is negative; that is, positively charged particles interact with them very strongly, whereas the electrostatic potential is larger and the negatively charged particles are easy to approach in the blue. Therefore, the vibration of C=O is easy to disturb.

**Figure 3:**
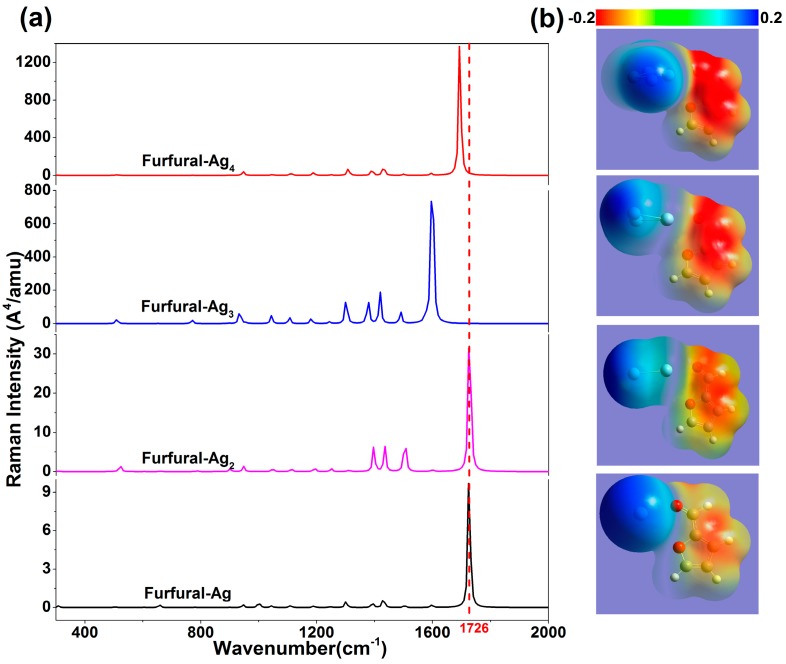
(**a**) The theoretical Raman spectra of furfural-Ag_x_ complexes with different atomic numbers (red dotted lines indicate characteristic peaks 1726 cm^−1^); (**b**) The static potential distribution of furfural-Ag*_x_* (*x* = 1, 2, 3, 4).

The Raman signal chemical enhancement properties of small silver clusters have always been the focus in experiment and calculation. However, for the surface enhancement effect, the physical enhancement is the main component, and it is difficult to determine whether the chemical enhancement exists during the experiment. Hence, the DFT is used to calculate the Raman enhancement of different Ag clusters on furfural. In Figure 3, the Raman spectra of furfural-Ag*_x_* (*x* = 1, 2, 3, 4) complex are given. Through comparative analysis, the variation of intensity of feature peaks is obvious with the increase of the silver atom, and the chemical enhancement effect of Ag clusters is stronger. For example, the Raman activity magnification has reached 120 times at 1726 cm^−1^ with an Ag cluster of furfural-M_4_. The reason for this includes two aspects: the change of molecular polarizabilities, and the increase in charge transfer with the transformation of Ag clusters. When the Ag clusters are applied on the furfural, the electronic charge redistributes in the complexes, resulting in changes in polarizabilities. In Table 2, the data show that static polarizabilities (α, the average value of α*_xx_*, α*_yy_*, α*_zz_*) and charge transfer of four complexes increase obviously, and the trend of variation is the same as with the change in Raman activities. The static potential diagram also produces corresponding changes as shown in Figure 3, and the electron enrichment region increases with an increasing number of atoms. It is also closely related to the redistribution of electrons. In addition, the Raman characteristic peaks at 1726 cm^−1^ are blue-shifted with increasing numbers, except for furfural-Ag_4_. The main reason for the blue shift (or red shift) of the Raman peak is the change of the corresponding chemical bonds, leading to the migration of electron clouds. Specifically, the change involves the transformation of interatomic bond force and distance. In Table 2, we examine changes in C=O bond distance, and find that the blue shift of the peak at 1726 cm^−1^ grows with the length of bond, whereas the red shift occurs when the complex is furfural-Ag_4_. These results are consistent with the above theory.

**Figure 4:**
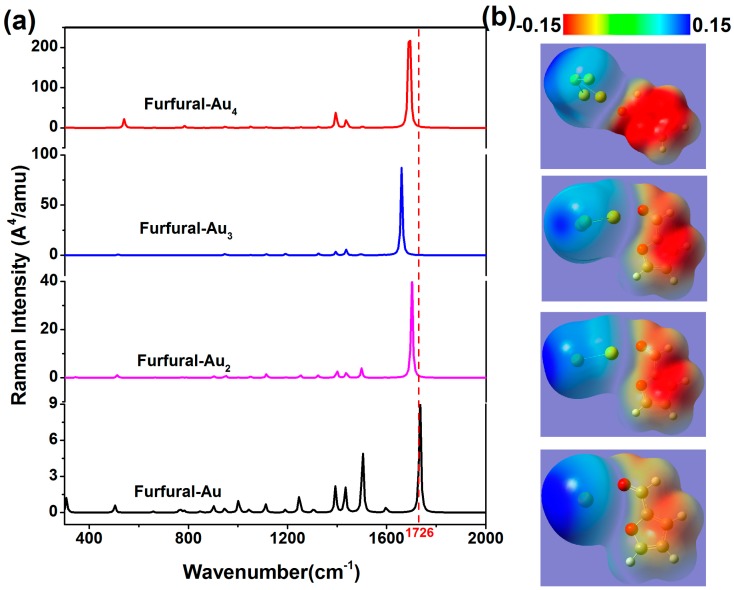
(**a**) The theoretical Raman spectra of furfural-Au*_X_* complexes with different atomic numbers (red dotted lines indicate characteristic peaks 1726 cm^−1^); (**b**) The static potential distribution of furfural-Au*_x_* (*x* = 1, 2, 3, 4).

**Figure 5:**
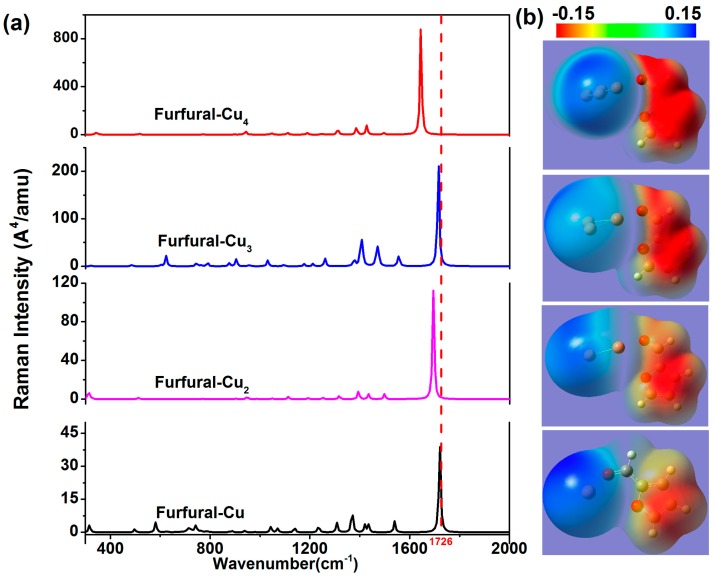
(**a**) The theoretical Raman spectra of furfural-Cu*_x_* complexes with different atomic numbers (red dotted lines indicate characteristic peaks 1726 cm^−1^); (**b**) The static potential distribution of furfural-Cu*_x_* (*x* = 1, 2, 3, 4).

In order to further study the influence of noble metal clusters on the Raman activities of furfural, the transformation of spectra with the changing of atomic species and number was calculated. The Raman spectra of furfural-Au*_x_* and furfural-Cu*_x_* complexes are shown in Figure 4 and Figure 5, and the number of atoms is varied. As mentioned earlier, the emergence of Au/Cu clusters leads to the redistribution of the electron density inside the molecule, resulting in a change in molecular polarizability and the Raman spectrum. In comparison to each other, the intensity of Raman activity is shifted with the increase in the number of atoms, and the trend of this change is the same as that of the former (The same phenomenon was also found in the Furfural-Ag*_x_*). In Table 3, we found that the change was also associated with the charge transfer. In contrast to Figure 3, the effect of chemical enhancement is also related to different kinds of atoms. The simulation results show that the SERS effect of silver atoms is mostly outstanding, and consistent with the experiment. Meanwhile, it is worth noting that the change of the peak at 1726 cm^−1^ varies with the number of noble metal atoms. For gold and silver atoms, the characteristic peaks at 1726 cm^−1^ occur blue shifted at *x* = 1 to 3 (*x* represents the number of atoms); moreover, the peak is red shifted only when *x* = 4. However, the occurrence of a red shift is different from the furfural-Cu*_x_*, and the characteristic peaks have a slight red shift for *x* = 3. In Table 2 and Table 3, the offset of the peak position corresponds to the change of C=O bond length. In addition, the chemical enhancement factors (EFs) for the Raman signals of furfural molecules with various metal atoms is also illustrated in Table 4, where the EF = (I_SERS_/N_SERS_)/(I_Raman_/N_Raman_)[27]. It is clear that the chemical EFs increase greatly as the number of atom increases, which can be explained by the increased charge transfers occurring in more atoms. Interestingly, for furfural-M_2_ (M = Ag, Au, Cu), EF_Furfural_-Cu_2_ > EF_Furfural_-Au_2_ > EF_Furfural-Ag2_, which is in agreement with the simulation results of previous reports [26,28]. However, for furfural-M_4_ (M = Ag, Au, Cu), EF_Furfural_-Au_4_ < EF_Furfural_-Cu_4_ < EF_Furfural_-Ag_4_, which is in agreement with the experimental results that indicated that the SERS enhancement of the Ag nanoparticle is greater than those of Au and Cu. Although the chemical enhancement of Cu atoms is greater than Au atoms, as shown in Table 4, the enhancement of Au nanoparticles is greater than Cu nanoparticles in SERS experiments. We understand this to be mainly due to the oxidation of Cu nanoparticles in air. This oxidation hampers not only the charge transfer in Cu atoms, but also the enhancement of the electromagnetic field. These results show that noble metal clusters have an influence on the Raman activity of furfural molecules, and the chemical enhancement effect caused by redistribution of charge is not negligible.

## 3. Theoretical Section & Experimental Part

All of the quantum chemical calculations were performed using the GAUSSIAN 09 program, and the molecules were constructed with GAUSSUAN VIEW 5.0. The ground state geometries of furfural-M*_x_* complex were optimized by density functional theory (DFT) method with the B3LYP functional. In all the calculations, the basis sets for C, H and O atoms were 6-311+g (2d, p), and the Ag atom was obtained using the pseudo-potential basis sets LANL2DZ. Moreover, the charge difference densities (CDDs) method was employed to visualize the distribution of electron density [29,30,31]. The static potential distribution (SPD) displayed the energy level of an electron [32,33,34].

All of the experimental data based on confocal laser Raman technology was set up in our laboratory. The platform was equipped with a low-nose diode-pumped solid-state laser (wavelength: 532 nm, power: 100 mW) and dispersive Raman spectrometer (ANDOR SR-5000i-C). A 50× long focal-length objective was used for laser convergence and signal collection. The pure furfural used in this work was provided by Chuanrun Lubricant Company, China. The sample was irradiated directly in a Petri dish. The integral time of the spectrometer was set at 5 s, and the accumulated integral was 6 times. In addition, the 600 L/mm grating and the 100m slit width of spectrometer were used to detect the sample.

## 4. Conclusions

In order to further understand the chemical enhancement mechanism in SERS, the DFT method was adopted to investigate the Raman spectra of individual furfural molecules and furfural-M*_x_* complexes. The simulated results were in good agreement with the experimental ones, except for a distinct difference at Raman peak 1726 cm^−1^. Our data suggests that this difference is mainly due to the lots of electron–hole pairs near the C=O bond (corresponding 1726 cm^−1^). In addition, the effect of Ag cluster size and adsorption position on Raman spectra was also analyzed in detail. The surface charge distributions indicate that the much stronger Raman signals with more metal atoms come from the greater numbers of electrons transferred to metal nanoparticle clusters and the increased polarizabilities of molecules. Finally, the influence of atomic species on molecular vibrational spectra was further calculated. These results showed that noble metal clusters have an influence on the Raman activity of furfural molecules, and the chemical enhancement effect caused by charge transfer was not negligible. Our studies strengthen the understanding of charge transfer effect in SERS of furfural molecules, which is quite important in the online monitoring of transformer aging process through SERS.

## Figures and Tables

**Table 1:** Raman vibration mode assignments of furfural molecules.

	Simulation (cm^−1^)	Experiment (cm^−1^)	Difference	Vibrational Plane	Vibrational Mode
1	494	503	−9	In-plane	C_3_=C_8_, C_8_–C_9_ symmetric bend
2	643	632	9	Out-plane	C_3_=C_8_, C_8_–O_4_ symmetric wag
3	761	757	4	In-plane	C_9_=O_11_ sway; C_8_–C_9_ stretch
4	884	884	0	Out-plane	C_1_–H_5_, C_3_–H_7_ synchronous sway; C_2_–H_6_ asynchronous sway
5	926	931	−5	In-plane	C_3_=C_8_, C_8_–O_4_ symmetric bend
6	1021	1021	0	In-plane	C_2_–H_6_, C_3_–H_7_ symmetric bend; C_2_–C_3_ stretch
7	1084	1079	5	In-plane	C_1_–H_5_, C_2_–H_6_ symmetric bend; C_1_–O_4_ stretch
8	1166	1159	7	In-plane	C_1_–H_5_ sway; C_1_–O_4_ stretch
9	1221	1225	−4	In-plane	C_3_–H_7_, C_1_–H_5_ asynchronous sway; C_2_–C_3_ stretch
10	1271	1279	−8	In-plane	C_8_–C_9_, C_8_–O_4_ asynchronous stretch; C_2_–C_3_ stretch
11	1372	1368	4	In-plane	C_9_–H_10_, C_1_–H_5_ synchronous sway
12	1398	1393	5	In-plane	C_8_–C_9_, C_8_–O_4_ asymmetric stretch; C_2_–C_3_ stretch; C_9_–H_10_ sway; C_2_–H_6_ sway
13	1474	1474	0	In-plane	C_1_–O_4_, C_8_–O_4_ symmetric bend; C_1_=C_2_, C_3_=C_8_ synchronous stretch; C_9_–H_10_, C_1_–H_5_ asynchronous sway
14	1567	1568	−1	In-plane	C_1_–O_4_, C_8_–O_4_ asymmetric bend; C_1_=C_2_, C_3_=C_8_ asynchronous stretch; C_2_–H_6_, C_3_–H_7_ synchronous sway
15	1726	1670	56	In-plane	C_9_–H_10_, C_8_–C_9_ synchronous stretch; C_9_=O_11_ asynchronous stretch

**Table 2:** The charge transfer, bond length (C=O) and polarizability of furfural-Ag*_x_* (α*_xx_*, α*_yy_*, α*_zz_* represent the polarizability of three axes respectively; α represents the polarizability of furfural molecules).

Atom Number (Ag)	Q (Furfural-Ag)/e	R (C=O)/Å	α*_xx_*/au	α*_yy_*/au	α*_zz_*/au	α/au
0	/	1.2137	95.731	65.934	38.447	66.704
1	0.034	1.2136	144.041	129.828	87.600	120.490
2	0.0428	1.2232	227.029	166.383	107.592	167.001
3	0.249	1.2390	377.191	238.700	180.260	265.384
4	0.284	1.2369	331.198	359.486	196.830	295.838

**Table 3:** The charge transfer and bond length (C=O) of furfural-M*_x_* (M = Au, Cu).

	Au		Cu	
Atom Number	Q (Furfural-Au)/e	R (C=O)/Å	Q (Furfural-Cu)/e	R (C=O)/Å
1	0.157	1.219	0.139	1.218
2	0.218	1.230	0.171	1.230
3	0.269	1.235	0.274	1.228
4	0.309	1.232	0.317	1.237

**Table 4:** Comparison of EF (Enhancement Factors) corresponding to three kinds of metal atom in different quantities (EF= (I_SERS_/N_SERS_)/(I_Raman_/N_Raman_)). (I_SERS_ and N_SERS_ represent signal strength and number of molecules in SERS, I_Raman_ and N_Raman_ represent signal strength and number of molecules in normal Raman).

Atom Number	1	2	3	4
Ag	1.74	5.68	132.24	246.46
Au	1.69	15.71	23.39	39.09
Cu	6.98	20.27	38.06	158.23

## Supplementary Materials

The following are available online at http://www.mdpi.com/2079-4991/7/8/210/s1, Figure S1: Raman spectra of pyridine–metal clusters simulated at the B3LYP/6-311+G** (C, N, H)/LanL2DZ(M) level. The unit of the differential Raman scattering cross is cm^2^ molecule^−1^ sr^−1^. (Figure comes from reference [2]), Figure S2: Experimental Raman spectra: The black line represent Raman spectra of pure furfural. The red line and blue line represent the Raman signals of furfural on gold or silver particles, respectively.
