# Supplementary material for: Charge Transfer Effect on Raman and Surface Enhanced Raman Spectroscopy of Furfural Molecules
Source: Nanomaterials (Basel). 2017 Aug 2;7(8):210. doi: 10.3390/nano7080210 (PMC5575692; doi:10.3390/nano7080210)
Supplement: Supplementary file 1 [file nanomaterials-07-00210-s001.pdf]

Supporting Information

Charge Transfer Effect on Raman and Surface Enhanced Raman Spectroscopy of Furfural molecule

Fu Wan[1*], Haiyang Shi[1], Weigen Chen[1], Zhaoliang Gu[1], Lingling Du[1], Pinyi Wang[1], Jianxin Wang[1], Yingzhou Huang[2*],

[1] State Key Laboratory of Power Transmission Equipment & System Security and New Technology, Chongqing University, Chongqing 400044, China; shihaiyang@cqu.edu.cn (H.S.); weigench@cqu.edu.cn (W.C.); gzl1989619@163.com (Z.G.); dulingling2014@163.com (L.D.); wangpingyi@cqu.edu.cn (P.W.); wangjianxin16@163.com (J.W.)

[2] Soft Matter and Interdisciplinary Research Center, College of Physics, Chongqing University, Chongqing 400044, China

* Corresponding author: fuwan@cqu.edu.cn (F.W.); yzhuang@cqu.edu.cn (Y.H.); Tel./Fax: +86-023-6567-8362 (F.W. & Y.H.)

1. Simulated Raman spectra of Pyridine-M$_2$ complex

Although the chemical enhancement of Cu atom is greater than Au one as shown in Table 4 , which is in agreement with the simulation results of previous reports[1-2], the enhancement of Au nanoparticle is greater than Cu one in comment SERS experiments. As we understood, this is mainly due to the oxidation of Cu nanoparticle in air. This oxidation hampers not only the charge transfer in Cu atom but also the enhancement of electromagnetic field.

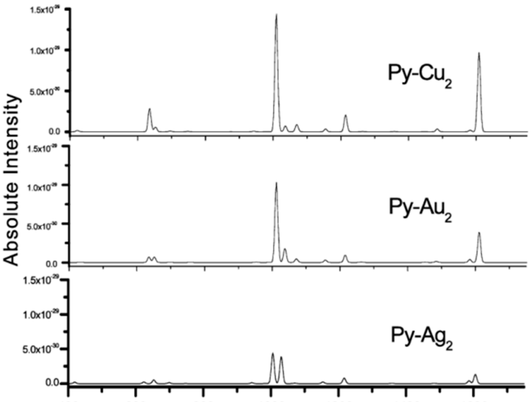

Figure.S1. Raman spectra of pyridine–metal clusters simulated at the B3LYP/6-311+G** (C, N, H)/LanL2DZ(M) level. The unit of the differential Raman scattering cross is cm^2 molecule^{-1} sr^{-1}. (Figure comes from reference [2])

2. Experimental Raman spectra of Furfural, Furfural-Au and Furfural-Ag

Silver Nanoparticles was prepared by heating reduction method with sodium citrate; The silicon wafer (0.5 cm ×0.5 cm) was cleaned respectively in deionized water and

anhydrous ethanol by ultrasonic for 20 min at room temperature, and dried by nitrogen. Then, the washed silver nanoparticles were added to the silicon wafer to evaporate naturally. Finally, the pure furfural solution was added to the enhanced substrate and detected by confocal laser Raman technology.

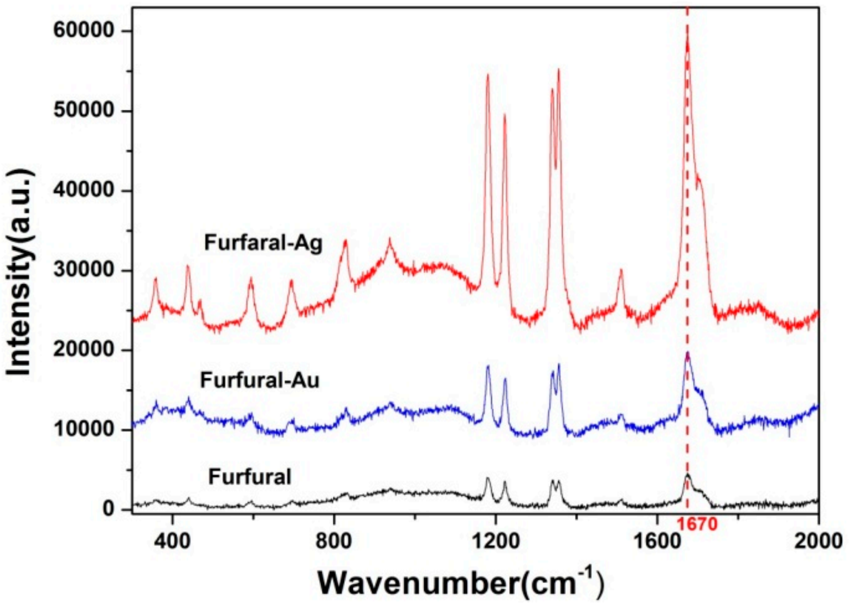

Figure S2. Experimental Raman spectra: The black line represent Raman spectra of pure furfural. The red line and blue line represent the Raman signals of furfural on gold or silver particles, respectively.

References

[1] Wu, D; Duan, S; Ren, B; Tian, Z. Density functional theory study of surface-enhanced Raman scattering spectra of pyridine adsorbed on noble and transition metal surfaces. J. Raman Spectrosc. **2005**, *36*, 533–540.

[2] Wu, D Y.; Hayashi, M.; Lin, S H.; Tian, Z.Q. Theoretical differential Raman scattering cross-sections of totally-symmetric vibrational modes of free pyridine and pyridine-metal cluster complexes. Spectrochim. Acta Part A Mol. & Biomol. Spectrosc. **2004**, *60*,137–146.
